# Novel Networked Remote Laboratory Architecture for Open Connectivity Based on PLC-OPC-LabVIEW-EJS Integration. Application in Remote Fuzzy Control and Sensors Data Acquisition

**DOI:** 10.3390/s16111822

**Published:** 2016-10-31

**Authors:** Isaías González, Antonio José Calderón, Andrés Mejías, José Manuel Andújar

**Affiliations:** 1 Department of Electrical Engineering, Electronics and Automation, University of Extremadura, Avenida de Elvas, s/n, Badajoz 06006, Spain; ajcalde@unex.es; 2 Department of Electronic, Computer Science and Automation Engineering, University of Huelva, Escuela Técnica Superior, Crta. Huelva-Palos de la Fra, Palos de la Fra, Huelva 21919, Spain; mjias@uhu.es (A.M.); andujar@diesia.uhu.es (J.M.A.)

**Keywords:** networked remote laboratory, data acquisition, programmable logic controller, fuzzy control, easy java simulations, OPC

## Abstract

In this paper the design and implementation of a network for integrating Programmable Logic Controllers (PLC), the Object-Linking and Embedding for Process Control protocol (OPC) and the open-source Easy Java Simulations (EJS) package is presented. A LabVIEW interface and the Java-Internet-LabVIEW (JIL) server complete the scheme for data exchange. This configuration allows the user to remotely interact with the PLC. Such integration can be considered a novelty in scientific literature for remote control and sensor data acquisition of industrial plants. An experimental application devoted to remote laboratories is developed to demonstrate the feasibility and benefits of the proposed approach. The experiment to be conducted is the parameterization and supervision of a fuzzy controller of a DC servomotor. The graphical user interface has been developed with EJS and the fuzzy control is carried out by our own PLC. In fact, the distinctive features of the proposed novel network application are the integration of the OPC protocol to share information with the PLC and the application under control. The user can perform the tuning of the controller parameters online and observe in real time the effect on the servomotor behavior. The target group is engineering remote users, specifically in control- and automation-related tasks. The proposed architecture system is described and experimental results are presented.

## 1. Introduction

The goal of communication networks is to share resources. Its use offers significant advantages in terms of reliability, enhanced resource utilization, reduced wiring, easier diagnosis and maintenance, and reconfigurability. Advances in Information and Communications Technologies (ICT) favor the use of these communication networks as data transmission speed increases continuously. Such ICT are also exploited for data storage based on the cloud. In fact, utilization of the cloud is an increasing trend in engineering, mechatronics and automation [1,2,3]. Another example is the development of the Internet-of-Things (IoT) [4,5].

**Figure 1:**
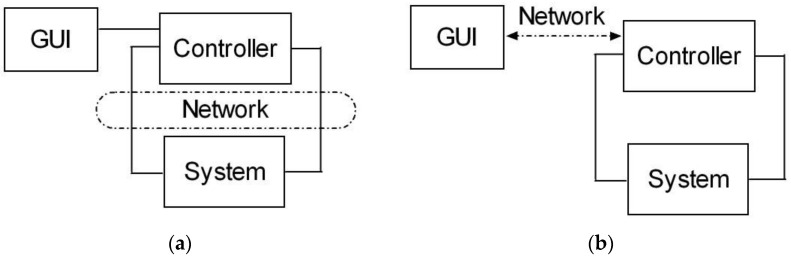
Schemes of: (**a**) NCS; (**b**) remote supervisory system.

From the point of view of control and supervisory systems, the utilization of communication networks can be considered in two aspects. Firstly, we consider a scenario in which the control loop is closed through the network. In such a case, the resulting approach belongs to the field of Networked Control Systems (NCS). In general, NCS are comprised of the system to be controlled and of actuators, sensors, and controllers whose operation is coordinated through some form of communication network [6]. On the other hand, the communication network can be integrated in the supervisory system to access the controller remotely. The purpose of this application is to perform the online parameterization of the controller and the monitoring of the process variables via a graphical user interface (GUI). Furthermore, the last application provides the possibility to access resources or facilities by remote users. Figure 1 shows the differences between these approaches. Figure 1a depicts the NCS scheme; as can be observed, the controller and the system interact through the network. In Figure 1b, the network serves to access the controller without intervention in the control loop.

In NCS the main problem to be solved is to overcome the constraints imposed due to the inclusion of the communication network in the control loop, namely the feedback data rate, fundamental performance bounds, network-induced delays or latency, control performance degradation, instability, and data losses. In the design process, the interaction of the control system with the network must be considered in order to use the communication resources effectively.

In the present work, the aim is focused on the access to the controller for supervisory tasks. The control is local and the user operates remotely the controller, as seen in Figure 1b. 

A particular case of the application of communication networks in remote supervision consists of the ability to carry out remote experiences. Remote Laboratories (RLs) fulfill this application and are becoming increasingly relevant in the engineering field. A RL can be defined as an environment whose function is to control a physical system remotely, aiming to teleoperate a real system, to perform experiments and to access sensors data over the network [7]. Both from an educational and industrial standpoint, the concept is the same: remotely operate plants. This involves pilot plants in the case of educational applications and full-scale plants for industrial environments. 

**Figure 2:**
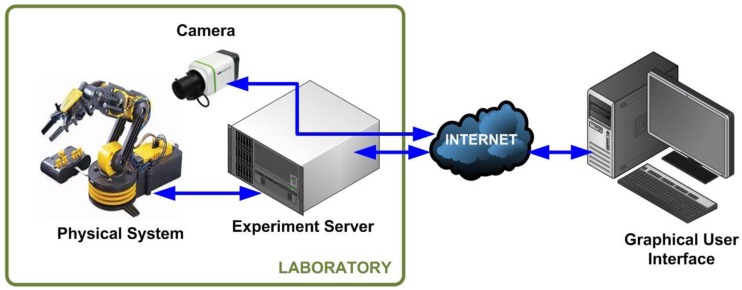
Scheme of a general engineering RL.

The typical scheme of a RL in engineering is depicted in Figure 2. It is composed of a physical system located in a laboratory equipped with Internet access. Such a system is usually a pilot plant based on an industrial plant, for example a robotic arm, and a Personal Computer (PC)-based controller to govern it in a local control loop (plant > sensors > signal processing > controller > drivers > actuators > plant). Generally an experiment server acquires the plant sensors’ signals and makes them available for remote access over the communication network. Finally, the user can observe and manage the behavior of the plant through a GUI. A camera provides video (and if interested also audio) to enrich the perception of the user, who has the possibility to access the RL essentially at any time and from any place.

This kind of architecture contributes to collaborative research since researchers located geographically distantly are able to develop control tasks over the same plant. Furthermore, RLs are expected to facilitate access to physical facilities to people under situations of disability, care responsibilities and so on [8,9,10]. On the other hand, developing countries are expected to take advantage of the benefits delivered by RLs [11,12]. 

In Spanish universities, several RLs have been developed for control and automation pilot plants with research and educational purposes: University of León [13,14], University of Huelva [15,16], National Distant Education University (UNED) [17,18], Polytechnic University of Madrid (UPM) [19], University of Alicante [20], Polytechnic University of Catalonia (UPC) [21,22], University of the Basque Country (UPV/EHU) [23], University of Deusto [24,25], University of Jaén [26], and University of Almería [27]. Even an online platform, UNILabs [28], has been created to establish a network of virtual and remote laboratories integrated by several of these Spanish universities. In the educational context, a deep analysis of RL application for control education can be found in [29,30]. For RLs oriented to Science, Technology, Engineering and Mathematics (STEM) education, current trends and features are addressed in [31]. The same authors propose a modular distributed architecture for RLs where users are able to design and share remote experiments for themselves [32].

Some trends in the field of RLs are: use of augmented reality [33,34,35,36], integration with the cloud [2], use of low-cost platforms such as the Raspberry Pi or Arduino [37,38,39] or integration of haptic devices [40]. 

Many of the RLs devoted to engineering and control have been developed using the software Easy Java Simulations (EJS), created by Francisco Esquembre [41]. It is a freeware, open-source tool written in Java to easily develop interactive virtual and remote laboratories delivered for users with no deep knowledge of the language [42]. Since its creation, it has been intensively used for virtual and remote laboratory design. It has been widely applied in university education and is continuously being enriched with new options and features. In the engineering field, two important releases of EJS enable a connection with external applications commonly used in laboratories, namely Java-Internet-LabVIEW (JIL) [43] and Java-Internet-Matlab (JIM) [44].

In addition, some useful tools have been developed to integrate devices and software packages with EJS. The most recently added ones are: the augmented reality component [45], the library for robotics and computer vision [20], Reader Apps for Android and iOS [46], the add-on for Moodle integration [47], the extension for collaborative support [48], Javascript use [49] and the model element for Arduino [16].

There are diverse options to carry out remote supervision. Some commercial packages such as NI LabVIEW [50] and Siemens WinCC include web-based access. A simpler possibility is to access the screen of a server placed in the monitored plant by means of Windows Remote Desktop or the open-source Virtual Network Computing (VNC) software. On the other hand, some authors have developed interfaces using different languages. For instance, Ko et al. [51] implemented a web-based laboratory with LabVIEW, HTML, Java and VB Script. Calvo et al. [23] use LabVIEW and Visual Basic to carry out this interface. In [52,53], the GUI is fulfilled using JavaScript and XML. Chevalier et al. [54] propose a RL based on C#, Matlab, Python and HTML, so a standard web browser can act as the client interface.

The main disadvantages of commercial solutions are their high cost and that usually they require that the user PC has specific computational resources, as a license for each user is necessary.

In this paper the design and implementation of a networked laboratory architecture devoted to controlling and monitoring a plant remotely are presented. This architecture integrates a programmable logic controller (PLC), the Object-Linking and Embedding for Process Control protocol (OPC) and EJS. The PLC carries out the controller functions and, depending on the plant, none, some or all signal processing, as well as driver and actuator functions. In our proposal, the PLC also plays an important role in the communication process. A LabVIEW application and the Java-Internet-LabVIEW (JIL) server complete the scheme for data exchange. Such integration can be considered a novelty in the scientific literature for RLs in control and automation engineering. This configuration allows the user to remotely interact with the PLC, overcoming the previously commented limitations.

EJS has been commonly used strictly with educational purposes; however, it has not been exploited out of such scope. In this work, EJS is used as a tool to develop the GUI of a networked laboratory with open connectivity to carry out remote experiences. 

A sample experimental application is developed to demonstrate the feasibility and benefits of the proposed approach. The experiment to be conducted is supervision and fuzzy control of a DC servomotor (henceforth referred to as DCSM). The GUI has been developed with EJS and the fuzzy control is carried out by our own PLC.

The remainder of the paper is organized as follows. In the following section a brief review of fuzzy logic, PLC and OPC in the context of RLs is shown. These elements constitute the core of the developed architecture; therefore, in order not to make the introduction section too large, we have preferred to carry out such a review in a separate section. Section 3 describes the elements of the RL architecture. Section 4 is devoted to exposing the developed architecture for data exchange, the fuzzy controller and GUI. Finally, the main conclusions and further works are outlined.

## 2. Brief Review of PLC, OPC and Fuzzy Control in the Context of RLs 

Since its formulation by Lotfi A. Zadeh half a century ago, fuzzy logic has grown and demonstrated suitability in multiple fields of science and engineering [55]. From the standpoint of control engineering, today fuzzy logic is a formalized discipline [56,57]. Fuzzy logic controllers (FLC) have many successful applications in industrial environments and in diverse technological contexts [58,59,60,61]. In fact, today, fuzzy logic is a tool widely used in the modeling of complex processes in multiple fields of science [62,63,64].

Regarding RLs, a few labs to apply FLC have been developed. Ko et al. [51] presented a web-based laboratory to apply Proportional-Integral-Derivative (PID) and fuzzy control to a coupled tank. It was implemented with LabVIEW, HTML, Java, VB Script and a data acquisition card (DAQ). Navarro et al. [65] designed a batch RL for position control of a DCSM under fuzzy PID control. It was based on a combination of HTML, Matlab and a DAQ. FLC as part of a RL for programming robots is reported in [66]; the HTML and C languages are used. In [67,68], a ball and beam system is subjected to four different control laws: PD, robust control, fuzzy control and reset control. The RL is developed with EJS, LabVIEW and a DAQ. 

PLCs are electronic devices extensively used to automate sequential processes. Reliable and robust operation have led them to be profusely applied in industrial processes and in other fields such as renewable energies systems, building automation, domotics and so on [69,70,71]. These devices are continuously increasing their capabilities according to technological advances in electronics and communications.

The PLC’s ability to support a range of communication methods makes it an ideal control and data acquisition device for a wide variety of industrial automation and facility control applications.

PLC networks provide you with a variety of networking options to meet specific control and communications requirements. Typical options include remote Input/Output (I/O), peer-to-peer, and host computer communications, as well as Local Area Networks LANs. These networks can provide reliable and cost-effective communications between as few as two or as many as several hundred PLCs, computers, and other intelligent devices. 

Many PLC vendors offer proprietary networking systems that are unique and will not communicate with another make of PLC. This is because of the different communication protocols, command sequences, error-checking schemes, and communication media used by each manufacturer. However, the trend of the manufacturers aims to share and establish standardized protocols to enhance the interoperability between their products. Some of the most widely used standardized protocols in industrial applications are PROFIBUS, PROFINET, MODBUS, Fieldbus Foundation, HART, ASi, LonWorks, DeviceNet, ControlNet, CAN Bus, Industrial Ethernet, etc.

**Figure 3:**
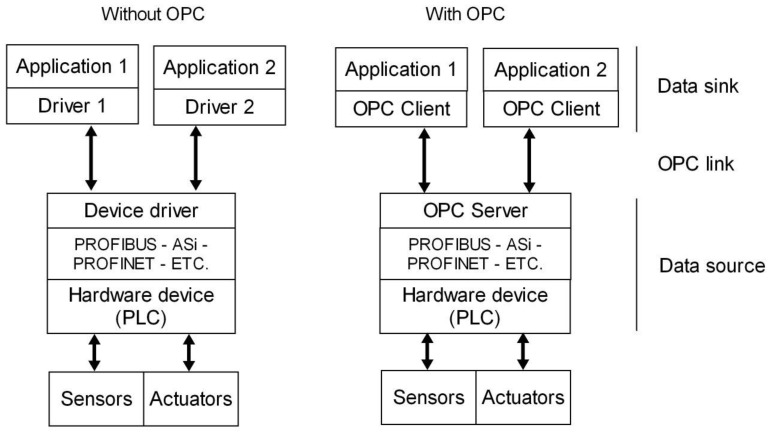
OPC protocol for data exchange in industrial environment.

However, it is possible to make different PLCs “talk” to one another or even to other hardware devices using a common language. The Object-Linking and Embedding for Process Control (OPC) assumes this role. The OPC protocol comprises seven specifications established by the OPC Foundation [72]. Based on client/server architecture, its goal is to provide open connectivity and reliable communication in automation. The intervention of specific drivers for each field device is eliminated by using the OPC standard. This way, hardware and software manufacturers provide a sole driver to connect the device to the OPC server and external applications are able to communicate with such a server without knowledge of the physical device. Figure 3 reflects the advantages of using the OPC protocol to exchange data. The hardware device acts as a data source through the OPC server, and the software applications play the role of data sinks (OPC clients) whereas the OPC link serves as a vehicle for data flow.

OPC technology is used throughout in industrial environments, but in the RLs context it has barely been used. For example, Schaf et al. [73] proposed a collaborative RL where the OPC is included to connect real or simulated devices such as PLCs. For the chemical engineering field, Klein and Wozny [74] developed a RL integrating Java and OPC communication. Calvo et al. [23] designed a RL combining LabVIEW, Visual Basic and the OPC interface to control a Ball and Hoop system. In [75], an approach based on a PLC and OPC link is proposed for a hands-on and remote PID tuning laboratory. Vadi and Bayndir [76] describe an OPC-PLC-based RL for a synchronous motor control experiment. The most recent work found was reported by Prada et al., 2015 [14], where the OPC protocol and LabVIEW are used in a RL to manage a flexible manufacturing cell.

PLCs have been previously validated as a useful resource for automation and control-devoted RLs. Besada-Portas et al. [77] implement a RL with an EJS interface and a TwinCAT PLC applying different controllers to a DCSM. In that case, the linkage between the PLC and the EJS was performed with a Java server application. Another example can be found in [52,53], where the GUI is implemented with JavaScript and XML. As stated by the authors, the use of PLC provides flexible connectivity at the back-end, allowing, through, it any kind of industrial plant device to be used in RLs.

The combination of PLC and fuzzy logic allows PLCs to be applied in systems where plant models are difficult to obtain due to nonlinearities, delays, etc. There are proprietary packages developed by some PLC manufacturers to program fuzzy algorithms, but their high cost and lack of flexibility make them uninteresting in Research and Development (R&D) scenarios.

In scientific literature, this powerful combination has been scarcely reported. Aydogmus [78] presents a fuzzy controller implemented with a Siemens PLC S7-200 to control a tank level. In [79], a method to develop fuzzy-PID controllers in a PLC for induction motors is presented. On the other hand, Bosque et al. [59] assert that the programming flexibility and the cost of PLCs contribute to the implementation of fuzzy control in industrial environments. Gizi et al. [80] report a combination of genetic algorithms and neural networks to tune a fuzzy PID controller in Matlab/Simulink which is applied using a Siemens PLC for voltage regulation in power generation systems.

As it is noted, the LabVIEW package is a versatile tool extensively applied for data acquisition and monitoring purposes in diverse technological and scientific fields [81,82,83,84,85,86,87,88,89,90]. Furthermore, LabVIEW is very often used in RLs devoted to control and automation. Some interesting examples are now mentioned. Stefanovic et al. [91] developed a RL entirely based on LabVIEW and a DAQ, whereas the plant under control is a set of four water-coupled tanks. Kuchirka et al. [92] reported an online laboratory to carry out control using a DCSM directly connected to a PC and a LabVIEW-based environment. In [93], a RL to carry out online experiments in the domain of mechanical material characterization is implemented using LabVIEW both for data acquisition and as a web server.

The plant under remote control used in our work is the well-known DCSM which has been used in diverse RL applications with successful results [22,65,77,92,94]. 

Regarding the inclusion of EJS, in [95] a RL for robotics, designed combining LabVIEW and EJS, is reported. With a similar framework, Chacón et al. [18] propose an architecture for the rapid development of RLs based on the joint use of LabVIEW, the EJS LabVIEW connector element, the JIL server and EJS. The plant under control is a coupled tank and the controller is a PID; the authors report a successful RL based on such architecture.

The principal contribution of the present work is the addition of the OPC protocol to the networked laboratory architecture in order to enable communication with PLCs. The combination PLC-OPC-JIL-EJS has not been previously reported in scientific literature. In addition, instead of a DAQ, the PLC performs the data acquisition and local control of the DCSM, whereas a LabVIEW program is in charge of data exchange. As a result, the distinctive features of the proposed novel network application are the integration of the OPC protocol to share information with the PLC and the application under control.

The usage of GUIs based on a web browser provides a platform-independent means for online access with high fidelity. However, in our proposal, a native desktop client based on EJS allows taking advantage of the capabilities of this tool, namely its features of being open source, the low programming skills requirement, the ability to integrate Java libraries, as well as its interactivity and user friendly interface.

In comparison with other architectures, those based on LabVIEW offer two options for remote access. On the one hand, the Remote Front Panel choice requires the installation of additional software, which is the NI Run-Time Engine in the remote user’s PC. On the other hand, if the web services option is selected, the effort needed to develop the HTML method Virtual Instruments (VIs) involves advanced knowledge of this communication. The RL frameworks that rely on high-level languages (HTML, Java, C, etc.) need deep programming expertise to be developed. Furthermore, most of the approaches in the revised literature are devoted to specific devices. Even they do not mention how the communication link between the physical plant and the experimental server is solved.

Our proposal is intended to overcome the aforementioned drawbacks. This way, a rapid development of RLs for automation and control applications is feasible. 

In conclusion, the developed architecture emerged as an effective solution to the need of performing remote experiences with open connectivity in the field of industrial control, automation and supervision.

The proposed approach offers the following advantages:

- Open connectivity with industrial field devices thanks to the use of the OPC.
- Portability due to the ability of EJS to run on the user’s PC with low requirements in terms of computational resources.
- Scalability to enhance the number of devices involved in the communication network.
- User-friendly and intuitive GUI.
- Useful information to provide an immersive remote experience.
- Flexibility to easily modify the code associated to both the LabVIEW program and the EJS GUI.
- Stability of the experience guaranteed by the LabVIEW’s reliable behavior.
- Functionalities improvements based on the EJS updates.
- Low cost for the user since EJS is a freeware tool.
- Real-time sensors’ measurement acquisition and transmission.

## 3. Developed RL

The experimental set-up of the RL is established in the Automation and Industrial Informatics Laboratory of the Electrical Engineering, Electronics and Automation Department at the Higher Technical School of Engineering (HTSE) of the University of Extremadura (Spain). 

**Figure 4:**
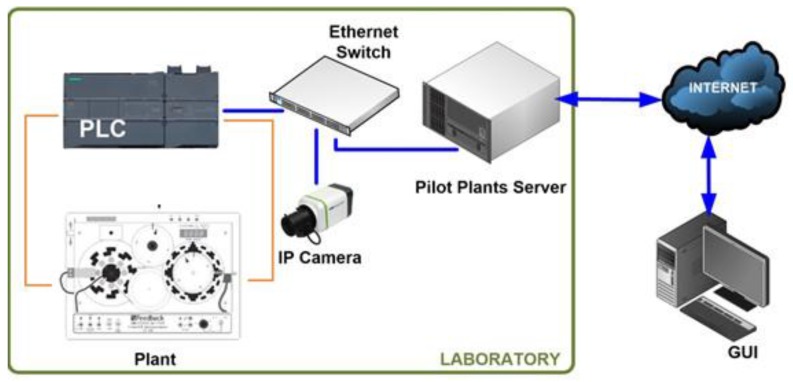
Schematic diagram of the proposed architecture.

The main components of the designed communication architecture are described in this section. The software packages involved in the system are also briefly commented on. Figure 4 shows the schematic diagram of the developed RL. As can be seen, the experiment server carries out tasks related to communication between the GUI and the PLC. Such a PLC governs the plant, i.e., the DCSM, and a camera provides video and audio feedback. These three components are connected to the LAN of the HTSE by means of an Ethernet switch, with the corresponding fixed Internet Protocol (IP) addresses.

The PLC, DCSM, pilot plant server and IP camera constitute the hardware subsystem.

The used PLC is the SIMATIC S7-1200, CPU model 1214C. With a modular and scalable design, this device belongs to Siemens low-end performance range and provides powerful resources in terms of calculation and communication capacities. It incorporates a microprocessor, an integrated power source, a slot for a memory card, 14 digital inputs, 10 digital outputs, two analogue inputs and an Ethernet/PROFINET interface. Furthermore, for the present application, an analogue output port is provided by a Signal Board module, SB 1232 AQ1. Such an output is used to apply the generated control signal to the DCSM.

The PLC acts over a plant which consists of the DCSM model Servo Fundamentals Trainer (33-001) of the manufacturer Feedback. It is composed of a mechanical unit (33-100) and an analogue unit (33-110). The first one contains a DC motor, an analogue tachogenerator, encoders, potentiometers, magnetic brake and other supporting electronics. The analogue unit is in charge of signals processing and a power supply (01-100) provides the required voltages. Both units are linked by means of a ribbon cable whereas the whole system can be Universal Serial Bus (USB)-interfaced with a PC. In the present application both the control and data acquisition are performed by the PLC so the DCSM is directly connected to it.

An IP camera transmits live video and audio from the plant to provide visual feedback to the remote user. This favors user immersion in controlling the plant. It is important to break the physical barrier which means that the operator (user) is not in the plant environment.

A PC, with OS Windows 7 Professional and Intel i7 CPU, acts as the pilot plant server (PPS) hosting the NI OPC server, the LabVIEW application, the JIL server and the software for the configuration of the PLC.

**Figure 5:**
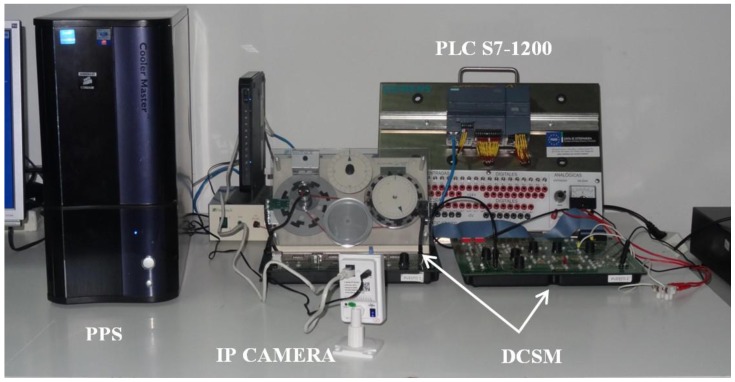
Experimental pilot plant of the developed RL.

Every component is connected to a unit power supply (UPS) that completes the system. In Figure 5 the pilot plant of the RL can be observed.

The software subsystem is composed of the following packages.

TIA Portal V13 (Totally Integrated Automation Portal) is the Siemens software for the PLC, Human-Machine-Interface (HMI) and network configuration. Particularly, the program STEP 7 performs the configuration and programming of the hardware and code tasks. In this case, it has been used to configure the network integration, I/O processing and FLC module. Such a module has been developed in the Ladder language for easy interpretation and adaptation to other devices.

The National Instruments LabVIEW (Laboratory Virtual Instrumentation Engineering Workbench) package is a high-level graphical language with powerful built-in functions. It is widely used in scientific and academic environments; it can even be considered the standard for instrumentation. The 2013 SP1 version and the Datalogging and Supervisory Control (DSC) module have been used to develop a VI which is in charge of transferring information available from the OPC server to the JIL module. This VI is named OPC-based Data Exchange Virtual Instrument (OPCDEVI). The OPC server has been created with the NI OPC Servers 2013 package.

As pointed in the Introduction, EJS is a freeware tool focused on discrete modeling and simulation, mainly oriented for users with low programming skills. Besides simulations, this simple and powerful tool allows designing environments for RL implementation. In our application, the EJS version used was released in September 2015. It is important to note that this software is continuously improved and updated by the contributions of several groups. 

On the side of the user, the proper operation of EJS requires an updated Java Runtime Environment. This feature is generally satisfied if the PC is frequently updated, something that can be expected for most of the users. However, the final user does not have to install any other software on his or her device. Additionally, a stable Internet connection is needed.

Detailed information about the integration of these packages is found in the next section.

## 4. Design and Implementation of the Developed Networked Laboratory Architecture

In this section the design, implementation and functionalities of the developed networked laboratory architecture are exposed. First, the base of the communication architecture is established; after that, the details of the sample application of this architecture (FLC running in a PLC) are given.

### 4.1. Communication between PLC and EJS

Usually the client/server architecture is used in most of the remote access applications such as RLs. In these ones, EJS implements both the client GUI and the information exchange with the server side. Such a server is commonly a PC-based control algorithm in conjunction with a DAQ device performing the local data gathering. In our developed scheme, data acquisition and local control are carried out by the PLC, whereas a LabVIEW application (OPCDEVI) interacts with both an OPC server and the GUI built with EJS.

As commented in the Introduction, to establish the data communication between EJS and LabVIEW VIs, a module called the Java-Internet-LabVIEW (JIL) server and the EJS LabVIEW connector element are used. As asserted by Chacón et al. [18], both tools provide an easy and effective means to build a RL with a three-layer architecture comprising a LabVIEW-based controller on the server side and an EJS -based GUI on the client side. 

The present proposal takes advantage of such an architecture and a fourth layer is added, performing the communication between the VI and PLC by means of the OPC protocol. Furthermore, the PLC is responsible for the control algorithm, not a software application. 

The proper use of the RL relies on the interaction of the user with the PLC memory positions to parameterize the controller and experiment. To this aim, the GUI contains a set of input fields that allow entering the desired values for the FLC parameters. The reference value for the speed of the DCSM is also written this way. Output fields are used to show the values related with sensors’ measurements and variables such as the actual speed, error and control signal. In addition, these values are represented over time to provide visual feedback information useful to the user. Therefore, the PLC memory has to be addressable and accessible for actions demanded by the GUI. The designed scheme satisfies this requirement.

**Figure 6:**
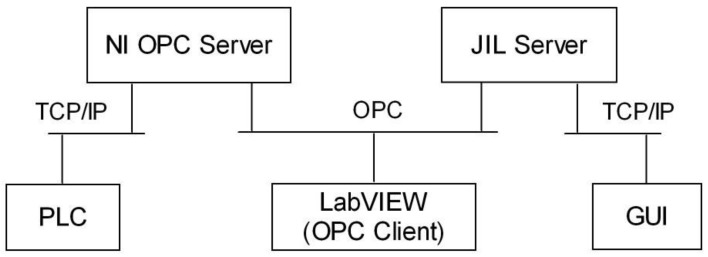
Interconnection layout between subsystems.

The data communication network acts as the backbone of the designed system. Data exchange between the PLC memory and RL interface has been developed using the structure of communications depicted in Figure 6. The signals involved in the control and monitoring of the DCSM are acquired and managed by the PLC. These ones are accessed by an OPC server so they are available for OPC clients. Such a role is played by a VI specifically created for exchanging data with the OPC server (OPCDEVI). The next stage consists of the JIL server, which establishes the connection between EJS and LabVIEW VI. This way, information flows between OPCDEVI and GUI, where the user interacts in real time with the remote experiment. Hereafter the involved parts are described sequentially, starting with the OPC server.

In general, the function of an OPC server is to access the data of a field device and make them available to OPC clients, i.e., software applications that perform operations with such data. In the present work, the NI OPC Servers 2013 package is used to create an OPC server. This one accesses the signals hosted in the PLC memory and makes them available for read/write operations commanded by the other components, particularly the OPCDEVI. 

**Figure 7:**
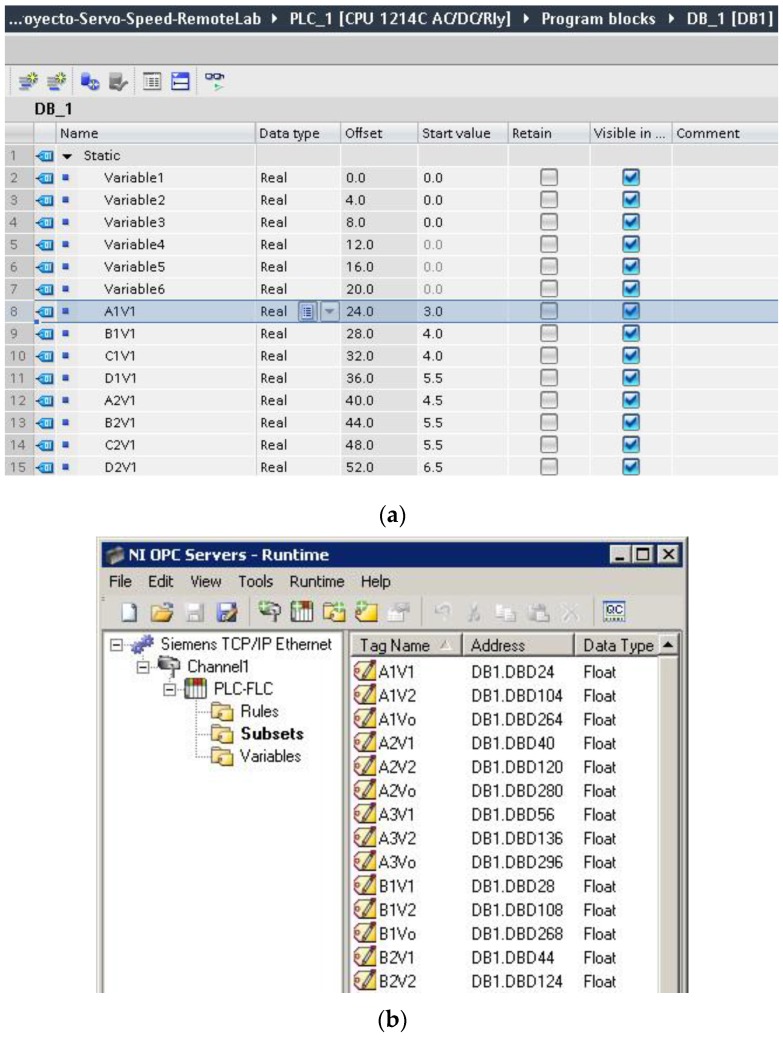
Details of configuration of variables and tags: (**a**) Variables in the PLC memory; (**b**) Tags created in OPC server associated with points defining the fuzzy subsets.

To do this, among other parameters to be specified, the most significant ones are the IP address and the device model, the used addresses in the PLC memory, the format of the data and the type of access. For the current sample application, three groups of tags have been created, one for I/O variables, another for the fuzzy subsets and the last one for the rules of the FLC. For instance, for the fuzzy subsets, the first tag corresponds to the point called A1V1. It is located inside a data block (DB1 of the PLC) in position number 24 with a float format, so its address is DB1.DBD24, as is shown in Figure 7a. On the other side, Figure 7b shows the OPC tags created for accessing these points.

The type of access, read only or read/write, depends on the intended target of the variable. The variables for read operations are: the actual speed of the DCSM, the error signal (calculated by the PLC) and the control signal generated by the FLC. The rest of the variables allow read/write access, namely: points for subsets definition, speed reference and indexes for the rule base. 

From the side of the OPC server, the OPCDEVI plays the role of the client. This VI has been designed to perform data exchange between the OPC server and JIL server. Therefore, it is only devoted to acquiring signals from the PLC through the OPC link and making them available for the EJS interface. 

**Figure 8:**
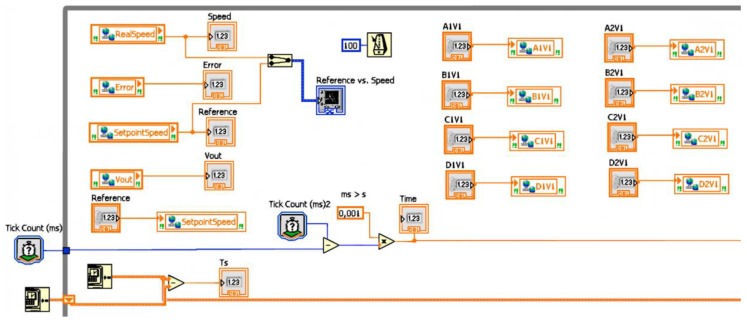
Graphical code for implementing data exchange in OPCDEVI.

The OPCDEVI consists of a while loop structure which executes readings and writings of variables. The corresponding code is shown in Figure 8. The LabVIEW DSC module has been used to configure this VI as the OPC client of the previously described OPC server. The variables shared by such an OPC server are added to the library of the VI, so controls and indicators are linked to them. Indicators are associated with signals that are read from the PLC, whereas controls are used to write the variables to the PLC. In addition, the variable Time is created to allow the representation of the process evolution in the interface. The JIL server imports the controls and indicators and therefore the gathered values are accessible for operations commanded from the GUI.

**Figure 9:**
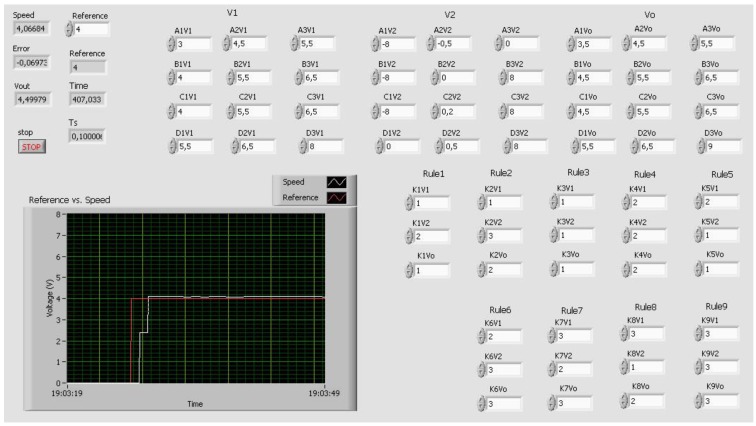
OPCDEVI for data exchange under operating conditions.

The sampling time has been configured to 0.1 s. This value is proper for the expected use of the RL; however, it can be easily changed in the VI code for other applications if required. Figure 9 shows the OPCDEVI running in the PPS.

Furthermore, OPCDEVI includes the code required to carry out data logging to support maintenance and surveillance tasks. To this aim, the most relevant variables of the system (reference, actual speed, error and controller output) and the Time variable are retrieved and stored in a lvm (LabVIEW Measurement) format file in the PPS hard drive. 

To sum up, this VI in conjunction with the JIL server plays the role of linkage between the PLC variables provided by the OPC server and the variables defined in the EJS-GUI.

Regarding the interoperability between EJS and LabVIEW VIs, it is provided by the JIL server. It consists of a VI devoted to exchanging data with other VIs based on the XML-RPC (eXtensible Markup Language—Remote Procedure Calling) protocol [18]. The EJS LabVIEW connector element is also required, so it has to be added and configured in the GUI.

The controls and indicators of the VI are published by the JIL server, so the EJS LabVIEW connector element is able to import and link them to the corresponding variables in the GUI. This way, the information of those controls and indicators is updated with the EJS. The configuration of the low-level communication is solved by these entities, making the development of the RL easier.

Both the NI OPC server and the JIL server are continuously running in the PPS to support the remote access. The OPCDEVI runs when it is called by the JIL server. This server attends the requests that the user sends through the connection/disconnection buttons of the GUI. User authentication is required to perform this step. In this stage, the GUI acts as the client of the JIL server.

**Figure 10:**
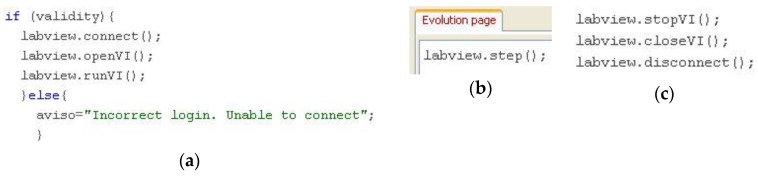
Details of methods for connection and disconnection with OPCDEVI: (**a**) Connection code; (**b**) Code in Evolution page; (**c**) Disconnection code.

The connection button basically executes the instructions for invoking the JIL server and running the VI. After that, this server opens the OPCDEVI, starting the data flow between the GUI and the PLC. Once the remote experiment has finished, the disconnection button stops and closes the VI and disconnects the JIL server. The required code is shown in Figure 10. Figure 10a corresponds to the code of the button, where the connection is initiated only if a Boolean variable associated with correct user identification is True. Otherwise, the instructions are not executed and a warning message is shown to the user. In the Evolution page (Figure 10b), the method labview.step () is called to periodically update the exchanged variables. No other code is required in this page. To conclude the remote access to the experimental system, the user has to push the disconnection button, whose code is shown in Figure 10c.

**Figure 11:**
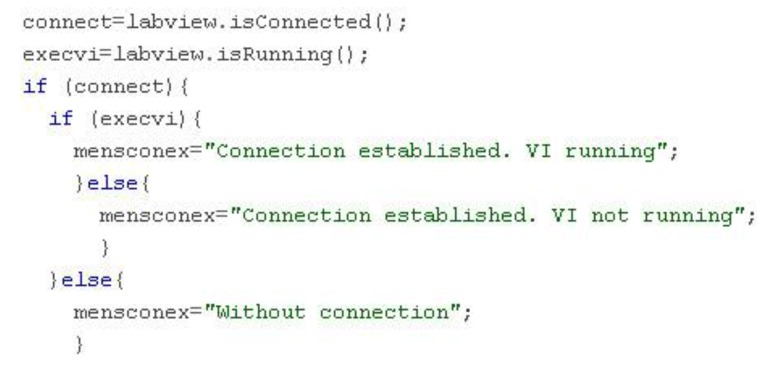
Code for connection-related messages.

There are two Boolean variables associated with the EJS LabVIEW connector element that provide information about the connection and VI state, isConnected () and isRunning (), respectively. A set of connection-related messages are generated in order to inform the user about both issues (Figure 11). 

**Figure 12:**
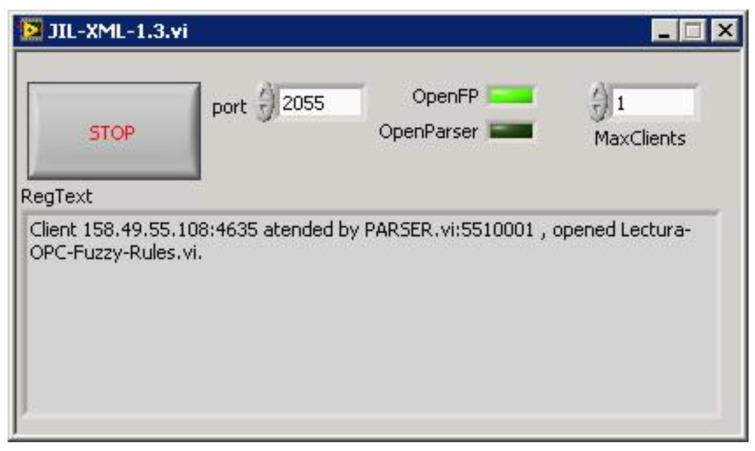
JIL server attending remote connections.

In Figure 12, the JIL server, after establishing a remote connection, can be seen. The Transmission Control Protocol (TCP) port is left at its default value, 2055. Another configurable parameter is the maximum number of simultaneous clients. In our application, the RL is designed to be used by one user at the same time, so this value is set to 1. For future applications, this server can accept multiple clients, allowing collaborative remote experiences.

**Figure 13:**
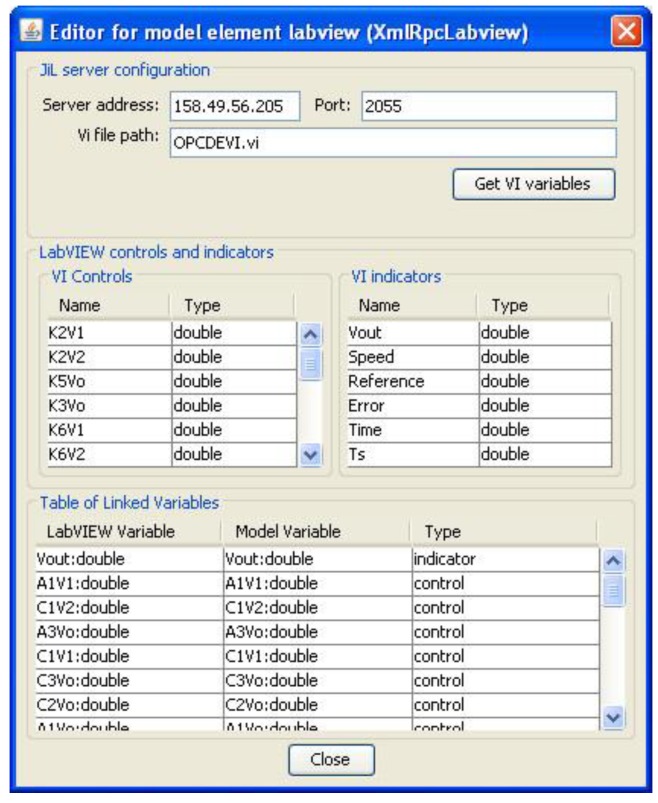
Wizard for configuration of EJS LabVIEW model element.

Figure 13 shows the configuration wizard for the EJS LabVIEW model element. The IP address of the PPS and the name of the developed VI are specified. Once the indicators and controls of this VI have been imported, the linkage between them and the EJS variables is carried out to establish the communication. To ease this linkage procedure, most of the variables defined in the GUI have received the same name as the OPCDEVI controls and indicators. 

As soon as the connection has been established, the values written in the GUI are continuously sent to the PLC memory as a consequence of the cyclic execution of the OPCDEVI. This feature allows the online parameterization of the controller by the user. A detailed explanation of the GUI is found in the next section.

**Figure 14:**
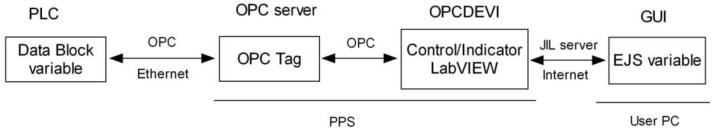
Stages followed by the exchanged data.

From the perspective of the exchanged data, Figure 14 synthesizes the sequence followed by the data to perform the networked remote pilot plant control according to the described architecture. Regarding the network connections, the path between the PLC and PPS belongs to the LAN of the HTSE. On the other side, remote access by the user implies that the exchanged data travel through the Internet to reach the PPS. 

The analysis of latency and network delays is not an aspect of this paper. However, it should be clarified that the delays in the network do not have a relevant impact in the control loop since the algorithm is locally applied by the PLC.

**Figure 15:**
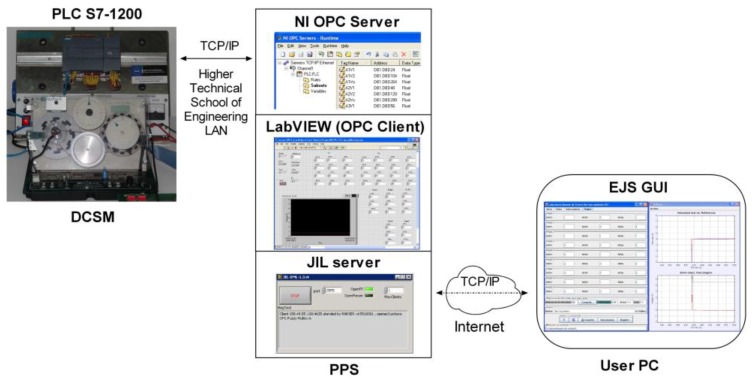
Scheme of the communication architecture.

Figure 15 graphically summarizes the involved software used in the communication architecture. 

### 4.2. Fuzzy Controller

Authors previously reported the design of the code for fuzzy control by means of the PLC and its experimental validation [96]. In the present work, such development is exploited as a remote experimental system. It must be noted that issues regarding FLC optimal design, stability or performance are beyond the scope of the goal of this work.

Fuzzy control consists of leading the process output to a desired value with control actions calculated according to a fuzzy description of this process, aiming to imitate the human reasoning process. A fuzzy controller comprises three stages: fuzzification, inference engine and defuzzification. The fuzzification of each natural value of the inputs consists of determining the degree of membership to each defined fuzzy set. The inference engine uses the fuzzy rules to process the input information and to generate the controller output. The defuzzification process converts the result of the fuzzy rules into a numeric or crisp value, non-fuzzy, which is the controller output signal. In the module for the PLC, each one of those parts has been solved by means of a subroutine, called function or FC.

The physical experiment to conduct through the RL is the speed control and monitoring of the DCSM. A FLC is implemented in the PLC so the user can adjust its parameters remotely. This obviously allows modifying the controller operation and thus the plant behavior.

The structure of the FLC has been made as simple and illustrative as possible. The type of the controller is Mamdani [97], the And method is Min, the implication operator is Min, the aggregation is Max and the defuzzification method is Centroid of area.

FLC parameters, which can be designed and modified by the users remotely, are membership functions of the variables and fuzzy rules. These are those that make up the logical structure and operation of the controller. The input signals are the voltages of the speed reference (or set point) and the error of the actual speed. Such an error is calculated by the PLC before executing the fuzzy module. The output signal is generated by the FLC and applied to the DCSM by means of an analogue output for the DCSM to reach the desired speed. 

The handled signals are the voltages corresponding to the magnitudes, i.e., the actual speed of the motor is calculated from the measured voltage. Nevertheless, we have considered that directly managing the voltages without conversion is illustrative enough for users’ comprehension.

**Figure 16:**
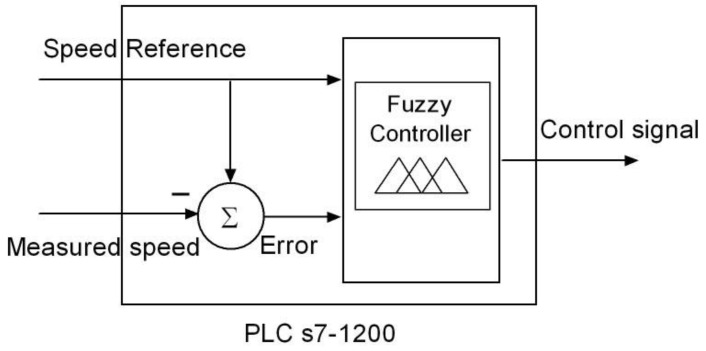
Schematic representation of I/O signals of the FLC.

Figure 16 depicts the scheme of the I/O signals of the FLC implemented by the PLC.

The design capabilities of the FLC in the PLC are higher than the options that users can modify. For example, the PLC accepts up to five fuzzy subsets per variable, but for the present application, only three are enabled. Another fixed parameter is the weighing factors; they have been fixed to a unitary value to simplify the FLC design.

The FLC parameters that can be modified are now described. The type of membership function can be defined between Trapezoidal, S, Z, Triangular and Singleton. Each one of them is a particular case of the Trapezoidal one, so the user chooses the preferred type by writing the points that define such a generic trapezoidal function. These four points are labeled as A, B, C and D for every subset of each variable. Three subsets are defined for both input and output variables. The linguistic labels are S1, S2 and S3, fixed by the developer to facilitate the nomenclature and comprehension of the subsets and rules. For instance, the points of the first subset (S1) of the first input variable (speed reference voltage, V1) are named A1V1, B1V1, C1V1 and D1V1. Similarly, the notation for the points of the second subset (S2) of the first variable is A2V1, B2V1, C2V1 and D2V1. These details appear in the initial tab of the GUI with an illustrative drawing, as can be seen in Section 4.3, Figure 16. It is evident that with this procedure to establish the subsets, the ranges of I/O variables do not need to be specified since they are implicitly expressed with the described points.

The rule base determines the reasoning procedure of the controller. In the PLC, the rule definition has been implemented by means of multiplexing operations, where an index indicates the subset associated with each variable in the rule. This way, the multiplexer selects the membership degree that corresponds to such a subset. Details can be found in [96]. An illustrative example of two rules is shown in Table 1, where Vo corresponds to the output variable of the FLC.

Since there are four points per subsets, three subsets per variable and three variables, the user can modify the values of these 36 parameters in the GUI. Furthermore, for the DCSM, nine rules have been formulated so users can modify another 27 parameters which define the rule base. Finally, the user sets the speed reference or the set point. Obviously, this value must be entered once the configuration of the fuzzy parameters has been fulfilled. If the user does not take this into account, this value simply has no effect until the FLC parameterization is completed.

It is important from the effective application perspective to note that online tuning of the parameters can be performed. Hence, users can modify the subsets and fuzzy rules to directly test the effect on the DCSM behavior in real time.

### 4.3. Overview of the EJS GUI

The GUI has been designed to be user-friendly and intuitive, affording continuous information about the process evolution, both numerically and graphically. In the design process of the GUI, two key aspects have been taken into account. On the one hand, the variables and configurations for data flow from and to the OPCDEVI (fuzzy parameters definitions, DCSM magnitudes) are considered. On the other hand, visual details of the interface are dealt. 

The GUI comprises four main parts: controller definition, real-time video, operative buttons and fields, and a graphical view of variables over time. These parts are contained in two separate windows, the main window and the graphics window. The main window is devoted to the first three parts and is divided in two distinct zones. The upper one is composed by four tabs, each one with a different role. The lower zone groups the DCSM-related variables and the operative buttons. The main window screen is shown in Figure 16, where the commented zones are highlighted.

The operative buttons implement the user authentication, connection/disconnection, play/pause and reset. On the other hand, a set of fields display warnings about the user login, messages about the connection state and the signals of the controlled system. Finally, an input field serves to enter the speed reference value. 

As seen in this figure, the described lower zone remains visible when the tabs are shifted, facilitating continuous access to this data. With this configuration, the user can easily change the tab without losing sight of this data. The user can see the graphics continuously and at the same time can change the tab to modify the parameters or view the real operation.

**Figure 17:**
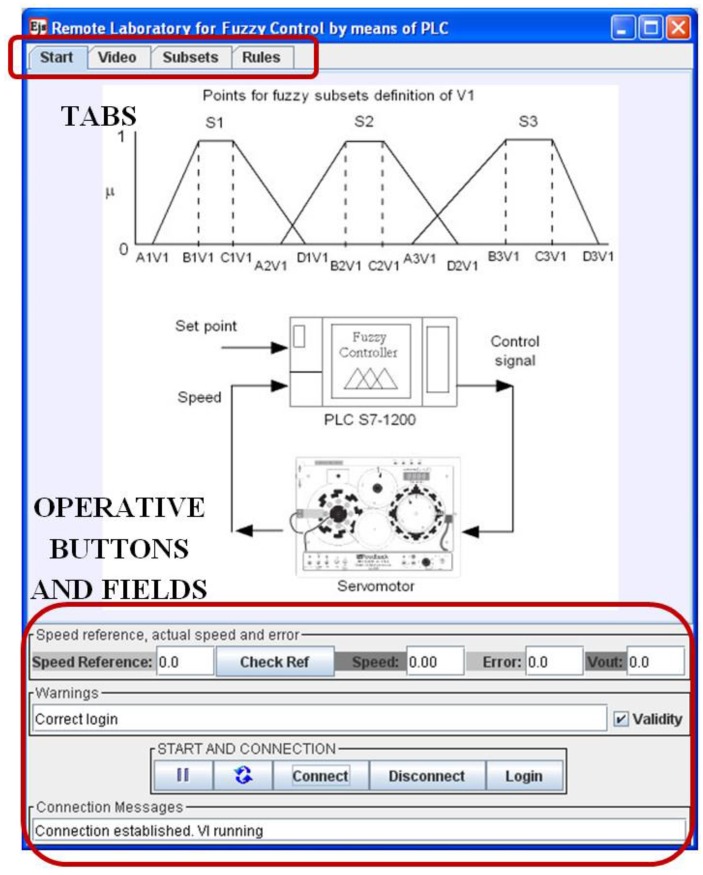
Layout of the GUI main window and initial tab.

Next, the upper zone is described. The first tab is the initial one and contains a draw illustrating both the fuzzy subsets’ nomenclature and the global scheme of the experimental set-up, as shown in Figure 17. This way, the user begins the RL session by getting a clear vision of the notation and the whole system.

The visual feedback information is provided in two ways: video/audio and graphics. The video/audio is received from the IP camera located in the physical laboratory, which is continuously transmitting. This contributes to a realistic perception of remote plant control. The second tab hosts the video image, as shown in Figure 17. As can be observed, the camera is oriented to focus on both the DCSM and the PLC, emphasizing the combination. 

**Figure 18:**
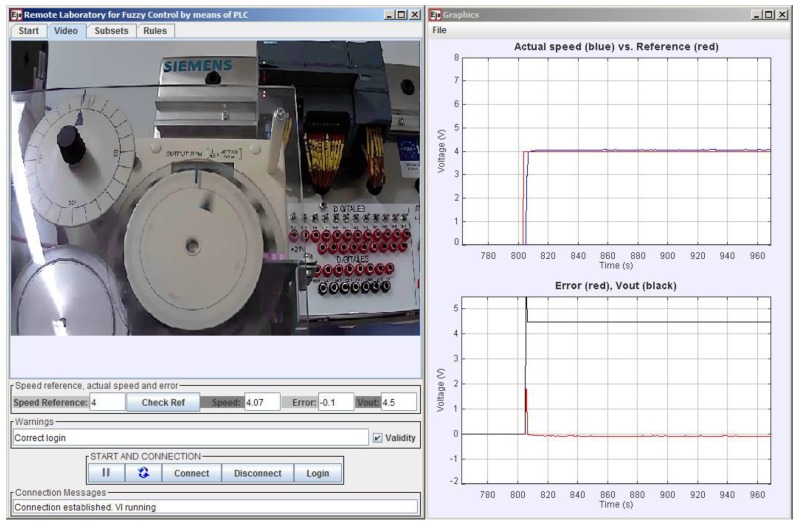
Visual feedback information provided by the GUI via video and graphics.

On the other hand, the graphics are responsible for showing the evolution of the variables over time, hosted in the second window. This graphics can also be observed in Figure 18. In these charts, the VI-generated variable Time is absolutely necessary for the representation of the process evolution. 

The first graphic is devoted to the simultaneous view of the reference and the actual speed voltage, located on the top. Error and Control signals are tracked below. A 4 V step signal (red color in the upper chart) is applied once the FLC has been defined. As can be seen, the DCSM follows the reference with a small error. Initially, this error reaches a high value until the steady state is achieved (red color in the lower chart). 

**Figure 19:**
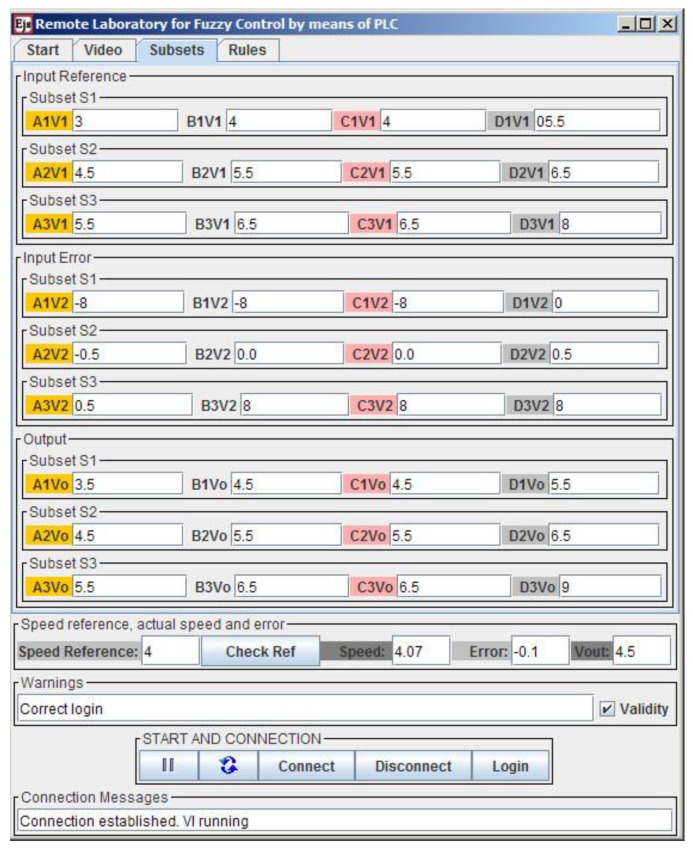
Tab for fuzzy subsets configuration.

The third tab is dedicated to entering the values corresponding to the points that define the membership functions of the input and output variables. The layout of this tab is as follows. There are three rows for each variable; each row contains four input fields to write the values of the points. These values must be introduced as real numbers. An example of suitable values for these points is shown in Figure 19.

**Figure 20:**
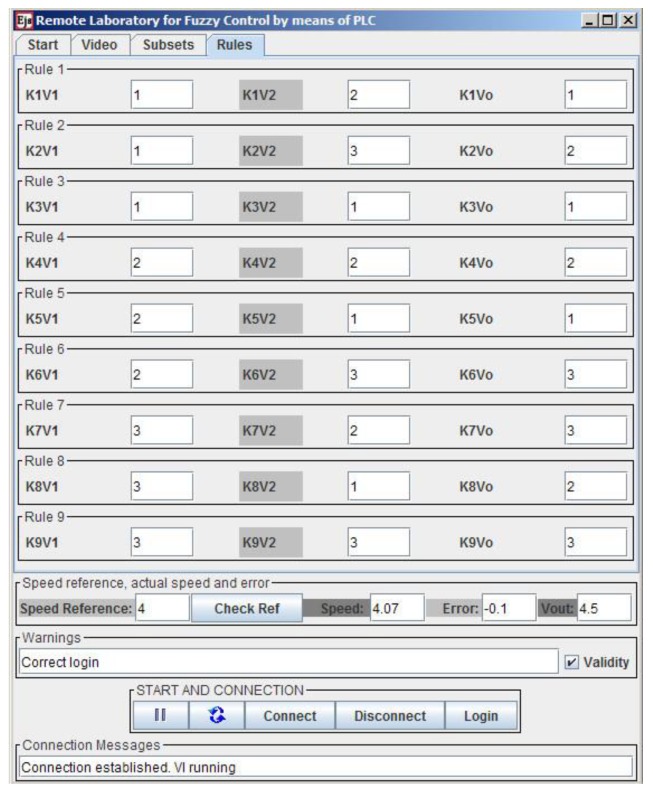
Tab for fuzzy rules definition.

The definition of rules is carried out through the last tab. There is a row for each of the nine rules. The user can modify the index values (integer numbers) corresponding to the input variables (antecedent) and to the output variable (consequent). This tab is shown with the values inserted during a remote connection in Figure 20.

All groups of parameters, subsets points and rules indexes can be adjusted online anytime, being automatically updated in the PLC. The corresponding effects are visualized in real time by means of the video transmission and the graphical tracking. 

The GUI is packaged into a ready-to-use portable file with the .jar format and a low weight of around 2 MB. Once downloaded, the user only has to execute it and then the interaction starts.

## 5. Conclusions

The development of a communication network architecture for RL integrating PLCs, the OPC protocol and the open-source EJS package has been presented. The RL is devoted to remote sensor data acquisition and controlling plants. A LabVIEW VI and the JIL server complete the communication scheme. 

The most outstanding contribution of the present work is the addition of the OPC protocol to enable communication with PLCs. The combination PLC-OPC-JIL-EJS has not been previously reported in scientific literature. 

A sample experimental application has been developed to demonstrate the feasibility and benefits of the proposed approach. The experiment to be conducted is the monitoring and fuzzy control of a DCSM. The GUI has been developed with EJS. The user can perform the tuning of the controller parameters online and observe the effect on the DCSM’s behavior in real time. 

A LabVIEW-based application, OPCDEVI, has been designed to carry out the information flow between the OPC and JIL servers. Note that the OPC-based layer leaves the field device aside from the rest of the communication system. This feature makes the modification of the field level easier. The presented approach has the advantages of high connectivity, flexibility, versatility and cost-effectiveness.

The GUI comprises two main parts: on the one hand, the definition and adjustment of fuzzy parameters; on the other hand, the visual tracking of the system, based on real-time graphics and video. A lot of effort was put on giving easy-to-use and user-friendly features to the GUI.

The communication architecture and the operation of the whole system have been proved and validated.

In concordance with open-source philosophy, all generated software code described here is available for the R&D community.

## Figures and Tables

**Table 1:** Example of indexes to define fuzzy rules in the PLC.

Rule n	Antecedent	Index of V1 for Rule n	Index of V2 for Rule n	Index of Vo for Rule n
1	If V1 is S2 and V2 is S3 then Vo is S1	K1V1 = 2	K1V2 = 3	K1Vo = 1
2	If V1 is S1 and V2 is S1 then Vo is S3	K2V1 = 1	K2V2 = 1	K2Vo = 3

## Abbreviations

The following abbreviations are used in this manuscript:

DAQ	Data Acquisition Card
DB	Data Block
DCSM	DC Servo Motor
DSC	Datalogging and Supervisory Control
EJS	Easy Java Simulations
FLC	Fuzzy Logic Controller
GUI	Graphical User Interface
HMI	Human-Machine-Interface
HTSE	Higher Technical School of Engineering
ICT	Information and Communications Technologies
I/O	Input/Output
IoT	Internet of Things
IP	Internet Protocol
JIL	Java-Internet-LabVIEW
JIM	Java-Internet-Matlab
LabVIEW	Laboratory Virtual Instrumentation Engineering Workbench
LAN	Local Area Network
LVM	LabVIEW Measurement
NCS	Networked Control System
OPC	Object Linking Embedded for Process Control
OPCDEVI	OPC-based Data Exchange Virtual Instrument
PC	Personal Computer
PID	Proportional-Integral-Derivative
PLC	Programmable Logic Controller
PPS	Pilot Plant Server
RL	Remote Laboratory
STEM	Science, Technology, Engineering and Mathematics
TCP	Transmission Control Protocol
TIA	Totally Integrated Automation
USB	Universal Serial Bus
VI	Virtual Instrument
VNC	Virtual Network Computing
XML-RCP	eXtensible Markup Language- Remote Procedure Calling
